# Moral distress, coping mechanisms, and turnover intent among healthcare providers in British Columbia: a race and gender-based analysis

**DOI:** 10.1186/s12913-024-11377-2

**Published:** 2024-08-13

**Authors:** Jorge Andrés Delgado-Ron, Muhammad Haaris Tiwana, Alice Murage, Rosemary Morgan, Simran Purewal, Julia Smith

**Affiliations:** 1 Faculty of Health Sciences, Simon Fraser University, 8888 University Drive, Burnaby, BC V5A 1S6 Canada; 2 Department of International Health, Bloomberg School of Public Health, Johns Hopkins University, 615 N Wolfe St Suite E8527, Baltimore, Baltimore, MD 21205 USA

**Keywords:** Moral distress, Intersectionality, Equity, Race, Gender, Healthcare providers, Quantitative methodology

## Abstract

**Background:**

This study explores intersectionality in moral distress and turnover intention among healthcare workers (HCWs) in British Columbia, focusing on race and gender dynamics. It addresses gaps in research on how these factors affect healthcare workforce composition and experiences.

**Methods:**

Our cross-sectional observational study utilized a structured online survey. Participants included doctors, nurses, and in-home/community care providers. The survey measured moral distress using established scales, assessed coping mechanisms, and evaluated turnover intentions. Statistical analysis examined the relationships between race, gender, moral distress, and turnover intention, focusing on identifying disparities across different healthcare roles. Complex interactions were examined through Classification and Regression Trees.

**Results:**

Racialized and gender minority groups faced higher levels of moral distress. Profession played a significant role in these experiences. White women reported a higher intention to leave due to moral distress compared to other groups, especially white men. Nurses and care providers experienced higher moral distress and turnover intentions than physicians. Furthermore, coping strategies varied across different racial and gender identities.

**Conclusion:**

Targeted interventions are required to mitigate moral distress and reduce turnover, especially among healthcare workers facing intersectional inequities.

**Supplementary Information:**

The online version contains supplementary material available at 10.1186/s12913-024-11377-2.

## Background

Intersectionality is a theoretical framework rooted in Black feminism that promotes social justice and equity through multiple-axis analysis. In her seminal work, “Demarginalizing the Intersection of Race and Sex: A Black Feminist Critique of Antidiscrimination Doctrine, Feminist Theory and Antiracist Politics,” Kimberlé Crenshaw [1] analyzed how interlocking systems of privilege and oppression (i.e., racism and sexism) shaped the experiences of racialized women based on the various identities they carried or were perceived to carry. Since its inception, intersectionality has evolved as a framework beyond the original categorical identities of gender and race and can help us understand how multiple social identities intersect and reflect heterosexism, classism, ableism, etc. [2].

Existing research on recruitment and retention in the Canadian healthcare workforce sheds light on the unfair and varied effects of intersectional inequities. This includes experiences of covert or overt prejudices during job applications, the work environment’s nature, and barriers to equitable opportunities [3–5]. Such inequities have significantly influenced the composition of the healthcare workforce in Canada. For example, while over 80% of healthcare workers (HCWs) are women, most work in professions like nursing and in-home or community care (90.1%), whereas the proportion of women physicians ranges from 40.2 to 49.7% depending on specialty [6, 7].

Similarly, while immigrants and People of Colour (POC) make up a growing portion of workers in many sectors of the healthcare system [8], they are overrepresented in front-line care work, which entails challenging tasks, such as bathing, feeding, grooming, changing diapers, cleaning bodily wastes, and administering medication [9]. These dynamics reflect and reinforce inequities related to compensation (for example, the median wage for a physician in Canada is $233,726 compared to community care aids for whom the median salary is $42,328) and power within health system decision-making [10, 11].

Intersectional research further requires researchers to engage in reflexivity, to reflect on our social positionality and to recognize our own biases and blind spots [12]. As academic researchers, we are particularly at risk of the privilege hazard, “the phenomenon that makes those who occupy the most privileged positions among us—those with good education, respected credentials, and professional accolades—so poorly equipped to recognize instances of oppression in the world” [13]. We try to mitigate this hazard by first acknowledging our privilege, and then grounding our analysis in intersectionality to identify and situate inequities within the current colonial and patriarchal context that structures Canadian health systems.

### Objectives

Moral distress is a concept that has developed out of the nursing literature to explain the ethical conflict healthcare workers’ experience when they are unable to provide the care they feel obliged to due to external constraints [14]. There are numerous definitions of, approaches to, and debates around the concept of moral distress [15]. We do not engage in these debates here, instead adopting Morley et al.’s [16] definition, which defines moral distress as occurring when there is a casual link between a moral event and phycological distress, and is supported by empirical research applying feminist interpretive phenomenology to explore nurses life experiences of moral distress [14, 16]. Determinants of moral distress include organizational/work environment, poor work satisfaction and engagement, feelings of disempowerment and lack of autonomy [17]. In further empirical studies, moral distress has been associated with burnout and an increased risk of anxiety and depression [18–20], and is a well-established contributor to healthcare worker attrition, explaining up to 40% of its variance among nurses [21].

Little moral distress research applies gender-based, intersectional, or other equity-based forms of analysis. While there is a growing feminist literature on moral distress, many of these papers draw on feminist approaches primarily to inform their methods or contextualize results, without sustained analysis of social positions beyond gender [16, 22, 23]. A recent literature review found 73 studies on moral distress that included data on race or gender, but none that applied intersectional theory [24]. This lack of intersectional approaches limits our understanding of how moral distress affects Canada’s diverse healthcare workforce, and how equity-based approaches might mitigate the effects of moral distress among those most affected.

Research on moral distress in the Canadian context has found moderate levels of moral distress among registered nurses, with moral distress related to ethical climate [25]. Research comparing across professions (among ICU HCWs) finds moral distress to be higher among non-physicians, compared to physicians, lower with age, and greater with years of experience among nurses [26]. The COVID-19 pandemic intensified and created new sources of moral distress among HCWs in Canada [27]. Heightened psychosocial distress among critical care physicians pushed a third of them to consider leaving the job. A high proportion of workers reported experiences of moral injury related to work conditions, including workload and conflicting demands, low control, low social support, and low rewards [18, 28]. Human resource shortages and system challenges have also been identified as contributors to moral distress among community healthcare providers [29].

Currently, the Canadian healthcare system faces a decreasing supply of HCWs, low retention, and challenging workplace conditions. There is a shortage of family doctors, and over two-thirds of nurses are considering leaving the profession [30]. Strengthening Canada’s healthcare workforce requires a greater understanding of moral distress to inform interventions to address this pervasive issue and prevent harm to healthcare workers and related attrition.

The current study applies intersectional analysis to explore to what extent moral distress levels explain inequalities in turnover intention among doctors, nurses, and in-home- or community care providers in British Columbia (BC), Canada, specifically focusing on the intersection of race and gender identity. Our overarching goal was to contribute to more equitable working environments. Therefore, we have shared preliminary results with participants who consented to follow-up, as well as with representatives engaged in Equity, Diversity and Inclusion work in unions and associations representing healthcare workers, requesting feedback and forming partnerships in order to develop recommendations informed by lived experience.

## Methods

Our observational, retrospective study integrates intersectionality theory into quantitative analysis and reports based on the Strengthening the Integration of Intersectionality Theory in Health Inequality Analysis (SIITHIA) checklist [31] (Supplementary file). We posed our research questions and developed our survey informed by previous research with women HCW working in British Columbia during the pandemic [32].

### Data sources

Details about sampling, recruitment, measurement, and data collection had already been described elsewhere [33]. In short, we conducted an online survey of healthcare professionals from all health regions of British Columbia between October and December 2022. Research participants were recruited through professional networks. Participants who identified as nurses, doctors, or in-home and community care professionals were eligible. We excluded responses who completed the survey in less than 3 min, which were considered bot or spam.

### Ethical considerations

This study was part of a research project approved by the Simon Fraser University (SFU) Research Ethics Board (30001218) and consistent with the principles of the 1964 Helsinki Declaration and its later amendments. The survey participants were informed about the research objective and were made aware that participation was voluntary and that they could withdraw from the survey at any time. We assured them of the confidentiality of their information. Only those who agreed to participate and consented were allowed to fill out the survey.

### Measures

Our data collection reflects intersectional principles that would allow for a meaningful comparison between the intersecting positions of two axes of marginalization: sexism and racism. Therefore, we created a variable concatenating gender (men or women) and racial identity (indigenous, person of colour, or white). We assumed men and white-identifying participants were the most privileged categories, informed by feminist and anti-racist literature [34]. While participants were offered gender-diverse options (two-spirit, non-binary, and transgender), we excluded those who selected these options because their low counts were not amenable to inter-categorical analysis. People of colour included those identifying as Middle Eastern or North African, African descent, Hispanic, South Asian, and Southeast or East Asian. We grouped them based on the ‘visible minority’ classification followed by Statistics Canada, as done in previous research [9].

The cross-sectional nature of the survey limited our ability to incorporate temporal contexts; however, we collected data on age (Under 30, 30 to 39, 40 to 49, or 50 or more), and country of birth (Canadian-born or foreign-born), which incorporate life trajectories. Profession (physician, nurse, or in-home/community care aid) was considered as a unit of social aggregation.

The independent variables that are hypothetically modifiable included the type of work arrangement (full-time or part-time/casual) and two scales: moral distress and coping strategies. The moral distress scale had seven items from the moral distress questionnaire that relate to system-level root causes and breakdowns in workers’ interactions with patients and families [19], and two additional items evaluating time management for care of patients and self-care. Similarly, we developed an eleven-item questionnaire about coping mechanisms/strategies [33]. For both scales, each item is a composite score that multiplies frequency (five levels from 0 or ‘never’ to 4 or ‘very frequently) and intensity of distress (from ‘none’ to ‘distressing’) or effectiveness of coping strategies (from ‘none’ to very effective’). Altogether, the moral distress questions added to an overall “moral distress score” (range: 0 to 144), which is the added sum of all nine items, whereas the “mitigation score” (range: 0 to 275) reflected the overall effectiveness of strategies to cope with moral distress. The full set of items for each questionnaire are available as supplementary material.

The main outcome of the study was turnover intention. Participants were asked ‘Have you ever left or considered leaving a clinical position due to moral distress?’ Those who answered ‘No’ were categorized as not exhibiting the outcome, while those who said ‘Yes’ were categorized as exhibiting the outcome, irrespective of whether they had left their positions or not.

### Analysis

#### Descriptive statistics

We described our sample using frequencies and proportions for our categorical variables and mean scores and standard deviations for numeric variables. Following the Public Health Agency of Canada [31] recommendations, we compared (a) non-modifiable and temporal characteristics across outcome groups and (b) turnover intention and its hypothesized determinants across professions (a social unit of aggregation). We used the chi-square test to compare categorical variables and the t-test to compare continuous variables.

Boxplots were used to explore the intra-group heterogeneity and the relationship between both scales and professions. Then, we estimated mean moral distress total scores and corresponding 95% confidence intervals (CI) for each identity group. We fitted an ANCOVA model that included the composite variable and profession as predictors. Subsequently, we used the ‘*emmeans’* R package [35] to compute marginal mean scores by intersecting identities, therefore adjusting indirectly for the professional group.

#### Modelling

We employed a classification and regression tree model (CART) to explore the dynamics between variables in the dataset. This approach was selected for its effectiveness in visually representing and interpreting complex relationships. We used the ‘*rpart’* package in R, to “predict” the likelihood of considering leaving due to moral distress based on modifiable and non-modifiable characteristics [36]. Given our exploratory goal, the entire dataset was used to train the model. The number of splits was set to 30 and the depth of the tree was capped at 6 levels to ease interpretability. The goal was to identify intersecting identities in the tree and how modifiable and non-modifiable characteristics played a role in the decisions of healthcare workers. A secondary objective was to untangle the interplay between identities and professional roles. All analyses were performed using R v 4.3.0.

## Results

**Fig. 1:**
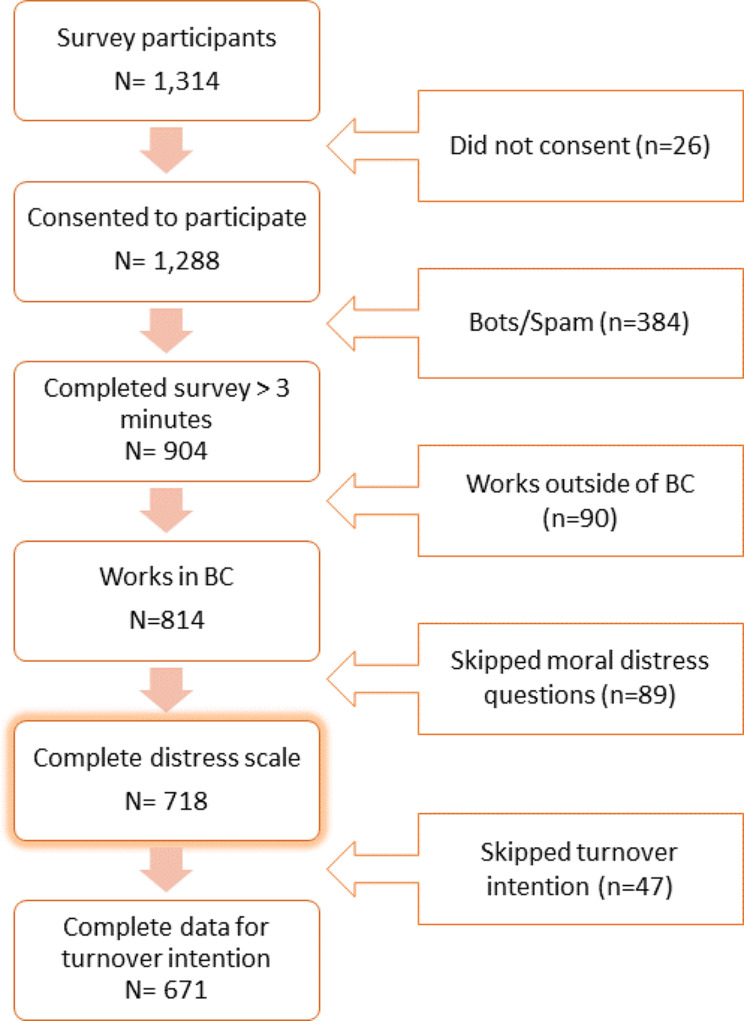
Flow chart of study participants; flow indicates analytic sample

Figure 1 shows the selection process for the final sample; 1,314 HCW participated in the survey. After excluding those who did not consent to the study, potential bots or spam, and those who did not work in BC, 814 HCWs were considered fully eligible (Fig. 1). 718 participants (or 88.20%) completed an adapted shorter version (9-item) of the moral distress questionnaire and included in our analytic sample (Fig. 1). 67.8% identified as women, and 34.7% identified as racialized (including indigenous and POC). Most of the respondents (93.45%) also provided data about their intention to leave their current position due to moral distress. Eligible participants who did not provide full outcome data were more likely to be foreign-born (*p* = 0.005), identify as POC (*p* = 0.008), and work as an in-home or community care aid (*p* = 0.003). We did not find statistically significant differences within the latter group between those who indicated their intention to leave work and those who did not.

### Turnover intention and non-modifiable characteristics

57.5% of the sample reported considered leaving due to moral distress. White women were significantly more likely to report the intention to leave work due to moral distress, while the opposite effect was observed in white men. This latter group had a significantly lower turnover intention (15.3%) than all other groups (range: 64.8–76.5%), which caused the proportion of intention turnover to be significantly different by gender and race, independently (not shown). Other non-modifiable characteristics significantly associated with turnover intention were Canadian-born and older than 50. Participants aged 40 to 49, conversely, were more likely to report they had not considered leaving (Table 1).

**Table 1:** Non-modifiable characteristics of the sample stratified by turnover intention

	Considered leaving due to moral distress		*p*-value*
	No (*n* = 285)	Yes (*n* = 386)	
Identity			< 0.001
* Indigenous men*	22 (8.3)	42 (11.6)	*1.000*
* Indigenous women*	19 (7.2)	36 (10.0)	*1.000*
* Men of colour*	6 (2.3)	15 (4.2)	*1.000*
* Women of colour*	20 (7.6)	43 (11.9)	*0.904*
* White men*	102 (38.6)	20 (5.5)	*< 0.001*
* White women*	95 (36.0)	205 (56.8)	*< 0.001*
Country of birth			
* Canadian-born*	245 (86.0)	308 (79.8)	0.048
* Foreign-born*	40 (14.0)	78 (20.2)	
Age group			< 0.001
* Under 30*	69 (24.2)	99 (25.6)	*1.000*
* 30–39*	87 (30.5)	150 (38.9)	*0.205*
* 40–49*	117 (41.1)	92 (23.8)	*< 0.001*
* 50 or more*	12 (4.2)	45 (11.7)	*0.005*

*p-values in cursive show Bonferroni correction

### Sample characteristics by social unit of aggregation

The sample was well-distributed among physicians (*n* = 178), nurses (*n* = 327), and in-home- or community care practitioners (H-CCP) (*n* = 213). After controlling for multiple comparisons, HCC-P in our sample had a significantly higher likelihood of identifying as indigenous men, being foreign-born, and working casually or under part-time contracts. No significant differences were found in terms of age or intention to quit. Nurses had a higher likelihood of identifying as white women and a lower likelihood of identifying as white men or indigenous men. They also concentrated a higher proportion of participants in the youngest (under 30) and oldest (50+) age groups. Nurses had significantly higher moral distress scores than physicians and H-CCPs (the p-value for the Tukey Honest Significant Difference test was < 0.01 in both cases). Nurses were significantly more likely to consider leaving the profession, while the opposite was true for physicians. Physician participants were mostly white men, more likely to be Canadian-born, aged 40 to 49, and full-time workers (85.4%). While physicians had the highest mean coping score, there were no significant differences across groups. Only 24.7% of physicians had considered leaving their position or had already left, compared to 61.3% of H-CCP and 72.7% of nurses (Table 2).

**Table 2:** Turnover intention and its hypothesized determinants stratified by profession

	Overall (*n* = 718)	H-CCP (*n* = 213)	Nurse (*n* = 327)	Physician (*n* = 178)	*p*-value
Considered leaving					< 0.001
* No*	285 (42.5)	75 (38.7)	85 (27.3)*	125 (75.3)*	
* Yes*	386 (57.5)	119 (61.3)	226 (72.7)*	41 (24.7)*	
Identity					< 0.001
* Indigenous men*	67 (10.0)	31 (16.3)*	18 (5.8)*	18 (10.6)	
* Indigenous women*	58 (8.7)	17 (8.9)	30 (9.7)	11 (6.5)	
* Men of colour*	21 (3.1)	9 (4.7)	8 (2.6)	4 (2.4)	
* Women of colour*	67 (10.0)	25 (13.2)	36 (11.6)	6 (3.5)*	
* White men*	128 (19.1)	31 (16.3)	8 (2.6)*	89 (52.4)*	
* White women*	329 (49.1)	77 (40.5)	210 (67.7)*	42 (24.7)*	
Country of birth					< 0.001
* Canadian-born*	588 (81.9)	147 (69.0)*	272 (83.2)	169 (94.9)*	
* Foreign-born*	130 (18.1)	66 (31.0)*	55 (16.8)	9 (5.1)*	
Age group					< 0.001
* Under 30*	173 (24.1)	50 (23.5)	104 (31.9)*	19 (10.7)*	
* 30–39*	252 (35.1)	79 (37.1)	110 (33.7)	63 (35.4)	
* 40–49*	226 (31.5)	63 (29.6)	70 (21.5)*	93 (52.2)*	
* 50 or more*	66 (9.2)	21 (9.9)	42 (12.9)*	3 (1.7)*	
Contract type					< 0.001
* Part-time or casual*	227 (31.6)	92 (43.2)*	109 (33.3)	26 (14.6)*	
* Full-time employee*	491 (68.4)	121 (56.8)*	218 (66.7)	152 (85.4)*	
Moral distress score (mean [SD])	60.37 [33.65]	48.68 [34.35]	74.19 [34.48]	48.95 [18.29]	< 0.001
Coping strategies score (Mean [SD])	78.08 [44.46]	76.81 [55.92]	75.94 [41.78]	83.32 [33.89]	0.210

* <0.05 after Bonferroni correction

### Profession, identity and moral distress

**Fig. 2:**
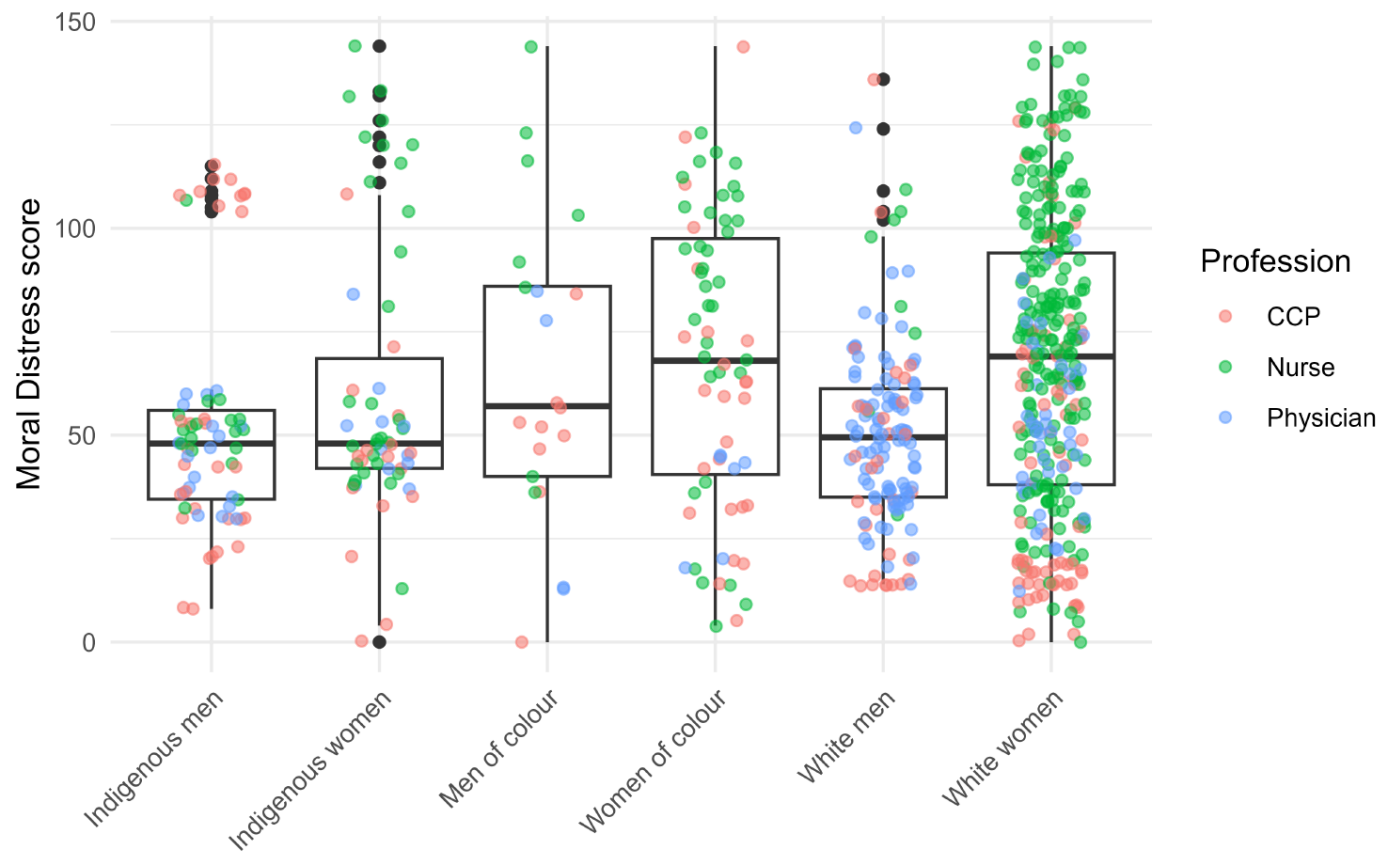
Distribution of the moral distress score by identities, classified by profession

Moral distress levels by identity, classified by profession, are shown as boxplots in Fig. 2. The interquartile range suggests higher variability for white women and POC men and women. The presence of outliers also highlights the professional role in the distribution of scores. For example, indigenous men with the highest distress levels work mostly as H-CCP, whereas indigenous women with the highest distress levels work mostly as nurses. We can also observe the relative absence of physicians among the outliers near the higher end of the y-axis (Fig. 2).

The mean moral distress scores ranked in descending order are shown in Table 3, along with the marginal scores, and corresponding 95% CIs. POC women had the highest mean score, significantly higher than white men. White women have significantly higher scores than both indigenous and white men. Such differences were no longer significant after adjusting for profession (Table 3).

**Table 3:** Mean moral distress score by identity, and marginal mean score adjusted by profession

Intersecting identity	Mean moral distress score	95% confidence interval (CI)	Marginal mean score^(a)^	95% CI
POC women	67.66	59.01–76.31	62.7	55.2–70.2
White women	67.57	63.74–71.40	60.0	56.3–63.7
POC men	65.05	47.81–82.28	63.9	50.8–77.1
Indigenous women	60.38	51.49–69.27	55.8	47.9–63.8
Indigenous men	52.99	46.26–59.71	54.7	47.3–62.0
White men	50.05	46.03–54.06	56.7	50.9–62.4

^a^ These estimated marginal means represent the mean values of the moral distress score within each intersecting identity subgroup, while statistically adjusting for the influence of profession

### Profession, identity, and coping strategies

**Fig. 3:**
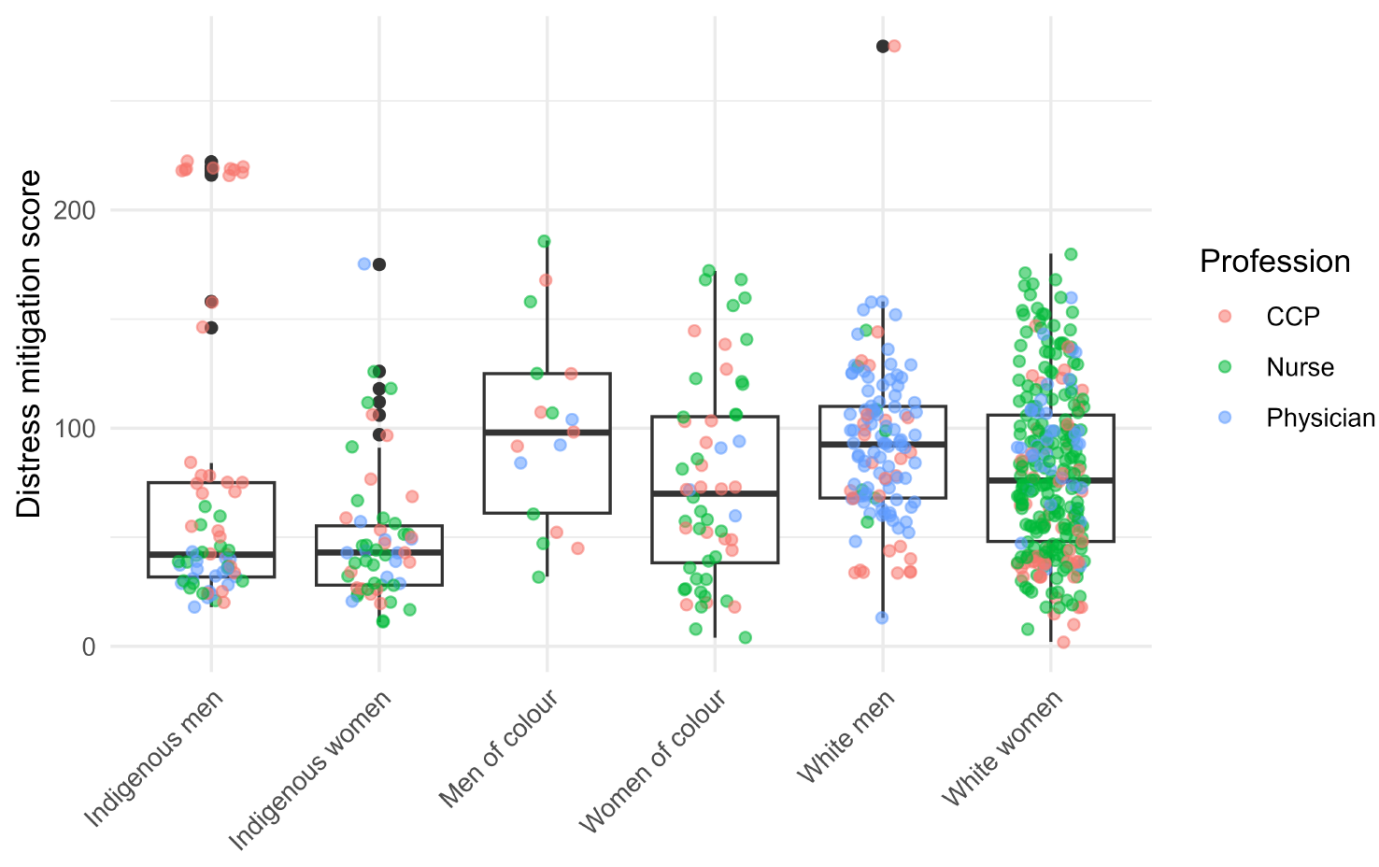
Mitigation score distribution by identities, classified by profession

Indigenous women had the lowest mitigation score compared to all other groups but Indigenous men. They also presented the least variability. Mitigation scores were also significantly higher for White men than White women (Fig. 3).

Unlike moral distress levels, the mitigation scores kept their original rankings and did not significantly change after adjustment, suggesting profession does not play a role in this association (Table 4).

**Table 4:** Mean distress mitigation score by identity, and marginal mean score adjusted by profession

Intersecting identity	Mean mitigation score	95% confidence interval (CI)	Marginal mean mitigation score^(a)^	95% CI
Indigenous women	49.09	40.44–57.75	49.1	37.8–60.4
Indigenous men	73.28	56.35–90.21	73.3	63.0–83.7
POC women	74.96	62.63–87.30	75.0	63.7–86.4
White women	80.14	75.76–84.52	80.2	74.6–85.7
White men	92.15	85.88–98.42	92.0	83.7–100.4
POC men	99.00	76.22–121.78	99.1	79.0–119.1

^a^ These estimated marginal means represent the mean values of the mitigation score within each group, while statistically adjusting for the influence of profession

Part-time and causal contract—more common among HCC-P and nurses—was also associated with turnover intention compared to full-time employment, which was common among physicians. People who considered leaving also had a significantly higher mean moral distress score (70.17 vs. 47.82) and a significantly lower mitigation score (71.93 vs. 86.02; *p* < 0.01 in all comparisons).

### CART model

**Fig. 4:**
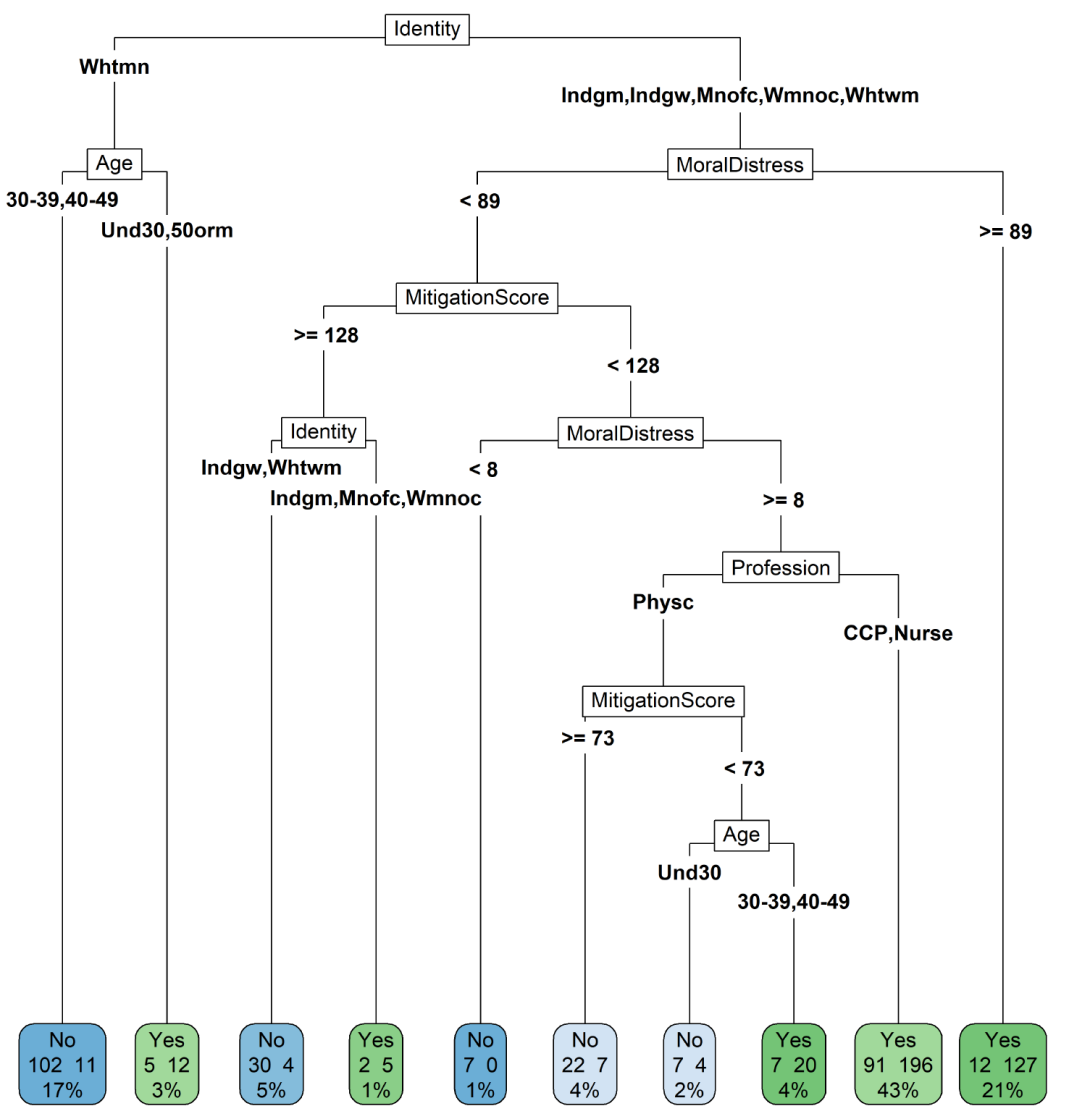
Classification and regression tree predicting turnover intention

The decision tree was split 9 times across six levels (Fig. 4). While the type of contract and country of birth were included in the model, they had a relative importance of 3% and 1% and did not play a role in splitting the nodes. The intersection of racial and gender identity was the most influential variable (42%), followed by moral distress levels (22%) and resilience scores (13%). Age and profession contributed 10% and 9%, respectively, to the model. For white men, neither the moral distress scale nor the resilience scale played a significant role in turnover intention. Instead, age—a non-modifiable factor related to life trajectory—played an important role. Among participants with other identities, 91% of people with a moral distress score equal to or above 89 intended to leave. When moral distress levels were lower than that and the mitigation scores were equal or above 128, most participants did not report the outcome (while there are differences in outcome by identity, the sample size is significantly small). Among participants who could experience either racial or sexist barriers and had reported moderate levels of moral distress (8 to 89) and low resilience score (< 128), profession played an important role: 68.3% of HCC-P and nurses had considered leaving. Among physicians who were women or racialized, those with very low mitigation scores (< 73) and between the ages of 30 and 49, had a high proportion of turnover intention (74.1%).

## Discussion

The high turnover intention among nurses (72.7%), and in-home and community health providers (61.3%) is telling and in-line with surveys by healthcare worker associations [37, 38]. Higher moral distress levels in nurses compared to physicians and H-CCPs have also been reported in cardiovascular surgery intensive care units in Canada [39]. Statistically adjustment by profession eliminated significant differences in moral distress scores across groups, suggesting that (a) such inequities could be partially explained by the extent to which each profession experiences moral distress, and (b) persistent inequality of access to professional roles. Finally, gender differences in moral distress also align with findings from surveys with healthcare workers during COVID-19 [18, 40].

Differential outcomes related to the mitigation scores of minoritized participants with elevated moral distress suggest the need for future research that incorporates intersectional approaches. For example, when analyzing the prevalence of turnover intention, White men had significantly lower turnover intention than all other groups. Nevertheless, gender-based or race-based analysis would have estimated significant differences for white people or all men, leaving white women and racialized men to “fall through the cracks.” Canada lacks detailed data regarding people with disabilities, race, or Indigenous identity of healthcare workers within health professions databases. Such omissions are concerning as they render experiences and representation of these workers invisible within data analysis and efforts for improvement [4].

The results from the CART model also imply that White men (most of whom work as physicians) based their turnover intentions on factors related to the life course and not moral distress. Conversely, elevated moral distress levels were a key factor in predicting the turnover intention for white women and indigenous and POC HCWs. Moral distress levels at the time of the survey likely reflected institutional constraints, many of which persist today [30] and are amenable to intervention.

Our research incorporates a novel approach to incorporate mitigation strategies in our understanding of moral distress. We found that availability and use of efficacious coping strategies predicted a lower turnover intention among White and Indigenous women. The same was not true for racialized men and POC women. However, the sample size for this specific subgroup was too small and more in-depth intersectional and context-specific research is needed to explore these suppositions.

### Strengths and limitations

Our study benefits from a significant sample size that allowed us to disaggregate data by gender and racial identity. To the best of our knowledge, no other quantitative moral distress studies has included such an intersectional analysis. Ours incorporates principles from intersectionality on qualitative research [31]. In doing so, we identified differential experiences based on both gender and racial identities that contributes to moral distress not evident when considering these identities separately. We explored the role of profession within these subgroups finding an inequality of opportunities that impacts moral distress. While descriptive statistics do not account for potential interactions or confounding effects between variables, CART modeling allowed us to handle nonlinear relationships, and complex interactions and to identify the relevance of each variable for particular groups.

Our study also had some limitations. Our measure of moral distress incorporates only a subset of items from the original questionnaire. However, our results align with previous moral distress surveys in assessing distress by gender and profession [18, 39, 40]. The analysis omitted gender identities, including non-binary respondents, due to the associated small sample sizes. Similarly, categorizing continuous variables like age into groups can lead to a loss of information and imposes an assumption of the same effect size within the age categories, which may not hold true. Furthermore, the data was collected towards the end of the pandemic which would have influenced the level of moral distress and turnover intention among HCWs. Finally, the study’s findings are specific to the sample population and may not be generalizable to all healthcare workers. POC and Indigenous men subgroups, for example, had small sample sizes and wider confidence intervals, increasing the probability of type 1 error in some analyses. Future research should work with these communities, potentially applying mixed methods to better understand their unique experiences.

Our team comprises diverse researchers, representing varying genders, races, and ethnicities, including those with healthcare work experience. However, we acknowledge that our team does not include anyone who identifies as Indigenous, and therefore, we are at risk of colonial and settler bias in our research. We have attempted to mitigate this by interpreting results related to Indigenous participants with humility and recognizing the colonial context. While our current work also does not significantly include qualitative interviews, it was informed by previous qualitative research [32].

## Conclusion

Moral distress is both an outcome and a contributing factor to the current human resource crisis in Canada. High distress scores, particularly among minoritized healthcare workers (the majority of the workforce), as well as their higher turnover intent, indicate an urgent need to address the root causes of moral distress, including inequitable access to professional roles. Systemic barriers, such as implicit biases in hiring practices, lack of mentorship, and limited opportunities for skill enhancement, prevent minoritized healthcare workers from advancing into higher-level positions or accessing professional development opportunities at the same rate as their counterparts. Differences across genders and races demonstrate how societal inequities permeate the healthcare sector, shaping varying levels of moral distress. For instance, Indigenous men in community care roles face particularly elevated levels of moral distress, reflecting unique challenges that necessitate targeted interventions. The implications for the healthcare system are significant: reduced diversity in leadership, hindered effective healthcare delivery, and increased turnover rates. Both immediate interventions and long-term structural changes are needed to mitigate the effects of moral distress on healthcare workers, particularly those already facing inequities, and prevent relate attrition within the healthcare system. This research highlights the urgent need to address systemic barriers within the healthcare sector and underscores the importance of tackling intersectional inequities to build a resilient healthcare workforce capable of meeting current and future challenges.

### Electronic supplementary material

Below is the link to the electronic supplementary material.

Supplementary Material 1

Supplementary Material 2

## Data Availability

Anonymized survey data is available upon reasonable request.
