# Supplementary material for: Moral distress, coping mechanisms, and turnover intent among healthcare providers in British Columbia: a race and gender-based analysis
Source: BMC Health Serv Res. 2024 Aug 13;24:925. doi: 10.1186/s12913-024-11377-2 (PMC11321194; doi:10.1186/s12913-024-11377-2)
Supplement: Supplementary file 1 — Supplementary Material 1 [file 12913_2024_11377_MOESM1_ESM.pdf]

Supplementary material

Strengthening the Integration of Intersectionality Theory in Health Inequality Analysis (SIITHIA) checklist

Study/ Report section	Item	Promising practice	Page
**Introduction**			
Background/ Rationale	1.	Provide a well-referenced definition of intersectionality theory, which alludes to its central principles*	**Error! Bookmark not defined.**
	2.	Describe inequalities that are consistently observed between population groups, which are assumed to be avoidable, as "unjust/unfair" and requiring action.	**Error! Bookmark not defined.**
	3.	Describe the known determinants of the outcome of interest that operate at, and above, the individual level.	**Error! Bookmark not defined.**
	4.	State and describe underlying assumptions underpinning the study, including a reflexivity* or positionality* statement from the research team.	**Error! Bookmark not defined.**
	5.	Integrate and summarize evidence developed through research and analysis that involve populations that are affected by the inequalities under study or forms of knowledge that have been under-represented in public health practice.	**Error! Bookmark not defined.**
Objectives	6.	Draw on, and describe literature and complementary theoretical frameworks (including those from outside the field of health sciences), as needed, to justify and frame the research questions and objectives.	**Error! Bookmark not defined.**
	7.	Explore one or more objectives relevant to intersectionality theory: Exploring to what extent observed health and social inequalities are explained by a given sub-set of characteristics or factors at the individual, community, or societal level.	**Error! Bookmark not defined.**
	8.	Engage with people and populations that are affected by the inequalities under study when establishing research questions and objectives.	**Error! Bookmark not defined.**
**Methods**			
	9.	Engage with populations that are affected by the inequalities under study, when designing the methods.	**Error! Bookmark not defined.**
Data source(s)		Where possible and relevant to the research question:	
	10.	Collect or use data that allow a comparison of outcomes across intersecting social positions*.	**Error! Bookmark not defined.**

	11.	Collect or use data that allow for an assessment of heterogeneity in determinants and outcomes* across social or spatial units of aggregation (e.g., schools, regions).	**Error! Bookmark not defined.**
	12.	Collect or use data that allow for an assessment of heterogeneity in outcomes across time (including temporal contexts based on calendar time, and inter-generational and lifecourse perspectives).	**Error! Bookmark not defined.**
	13.	Collect or use data that allows for an assessment of independent measures that are hypothetically modifiable, and therefore amenable to intervention.	**Error! Bookmark not defined.**
	14.	Collect or use qualitative data (e.g., using interviews, focus groups, open-ended survey questions, program evaluations, etc.) to complement quantitative data sources, in a mixed-methods research design approach.	-
Measures		Where relevant to the research question, operationalize independent measures that enable an assessment of outcomes across:	
	15.	Two or more axes of marginalization;	**Error! Bookmark not defined.**
	16.	Units of social or spatial aggregation or clustering;	**Error! Bookmark not defined.**
	17.	Temporal contexts (including contexts based on calendar time, and inter-generational and lifecourse perspectives).	**Error! Bookmark not defined.**
	18.	Operationalize and utilize independent measures that are hypothetically modifiable, and therefore amenable to intervention.	**Error! Bookmark not defined.**
	19.	Describe assumptions about the relationship between study measures, including the assumed direction and temporal ordering of associations, using a causal map or Directed Acyclic Graph*.	-
	20.	Describe assumptions about the broader social phenomena that measures are assumed to capture or represent.	**Error! Bookmark not defined.**
	21.	Describe and justify selected reference categories.	**Error! Bookmark not defined.**
Analysis	22.	Select/design analyses according to study objectives.	**Error! Bookmark not defined.**
	23.	Assess both absolute and relative inequalities between groups.	**Error! Bookmark not defined.**
	24.	In regression-based analyses, use a parsimonious set of adjustment variables based on the causal map described (Item #19).	NA
	25.	State and test underlying analytic assumptions using sensitivity analyses.	**Error! Bookmark**

			**not defined.**
	26.	Where relevant to the research question, analyze qualitative data, using methods most appropriate for the study's objectives.	NA
**Results**			
	27.	Present and discuss determinants, outcomes, and inequalities therein, stratified by i) relevant sub-groups, ii) units of space, iii) units of time.	**Error! Bookmark not defined.**
	28.	Present and interpret effect modification results, distinguishing between additive and multiplicative interaction.	NA
	29.	If available and relevant for the research question, present the results of the mixed-methods or qualitative analyses that were performed (Item #26), including key illustrative quotations.	NA
**Discussion**			
	30.	Engage with populations that are affected by the inequalities under study, for the review and interpretation of findings.	-
Findings	31.	Refer to principles of intersectionality theory when interpreting the plausible mechanisms explaining results.	**Error! Bookmark not defined.**
Implications	32.	Describe the implications of the study for public health practice, as well as policy and systems change.	-
	33.	Describe the implications of the study for the potential population targets of intervention (e.g., universal policy, targeted/proportional universalist policy).	**Error! Bookmark not defined.**
Limitations	34.	Describe how key/core principles of intersectionality were or were not integrated in the study.	
	35.	Describe any limitations of data sources (including statistical power), measures and analyses, and their implications.	**Error! Bookmark not defined.**
	36.	Include reflexivity about the power invested in (and reproduced by) the methods used.	
* Please refer to the report's Glossary section for definitions			
