# Supplementary material for: Moral distress, coping mechanisms, and turnover intent among healthcare providers in British Columbia: a race and gender-based analysis
Source: BMC Health Serv Res. 2024 Aug 13;24:925. doi: 10.1186/s12913-024-11377-2 (PMC11321194; doi:10.1186/s12913-024-11377-2)
Supplement: Supplementary file 2 — Supplementary Material 2 [file 12913_2024_11377_MOESM2_ESM.docx]

#### Moral distress questionnaire

Moral distress occurs when care providers cannot carry out what they believe to be ethically appropriate actions because of constraints or barriers. If you have experienced the situations listed, they may or may not have been morally distressing to you. Please indicate how frequently you have experienced each item over the past year (since October 2021). Also, rank how distressing these situations are for you. If you have never experienced a particular situation, select “0” (never) for frequency. Even if you have not experienced a situation, please indicate how distressed you would be if it occurred. Note that you will respond to each item by checking the appropriate column for two dimensions: Frequency and Intensity of Distress.

|  | **Frequency** | | | | | | **Level of Distress** | | | | |
| --- | --- | --- | --- | --- | --- | --- | --- | --- | --- | --- | --- |
|  | Never Very  frequently | | | | | | None Very  distressing | | | | |
|  | 0 | 1 | 2 | 3 | 4 | 0 | | 1 | 2 | 3 | 4 |
| 1. Been required to care for patients whom I do not feel qualified to care for. |  |  |  |  |  |  | |  |  |  |  |
| 1. Watched patient care suffer because of a lack of provider continuity. |  |  |  |  |  |  | |  |  |  |  |
| 1. Been required to work with other care providers who are not as competent as patient care requires |  |  |  |  |  |  | |  |  |  |  |
| 1. Witnessed low quality of patient care due to poor team communication. |  |  |  |  |  |  | |  |  |  |  |
| 1. Been required to care for more patients than I can safely care for |  |  |  |  |  |  | |  |  |  |  |
| 1. Experienced compromised patient care due to lack of resources or equipment |  |  |  |  |  |  | |  |  |  |  |
| 1. Experienced lack of administrative action or support for a problem that is compromising patient care. |  |  |  |  |  |  | |  |  |  |  |
| 1. Lacked the time to provide the care the patient needs. |  |  |  |  |  |  | |  |  |  |  |
| 1. Lacked the time to provide the self-care I need |  |  |  |  |  |  | |  |  |  |  |

#### Mitigation strategies questionnaire

Please indicate how frequently you have employed the following strategies to help cope with effects of distress over the past year (since October 2021). Also, rank how effective these strategies are for you. If you have never applied a particular strategy select “0” (never) for frequency. Even if you have not applied a strategy, please indicate how effective it would be.

|  | **Frequency** | | | | | **Effectiveness** | | | | |
| --- | --- | --- | --- | --- | --- | --- | --- | --- | --- | --- |
|  | Never Very  Frequently | | | | | Not Very  Effective | | | | |
|  | 1 | 2 | 3 | 4 | 5 | 1 | 2 | 3 | 4 | 5 |
| C.1 Exercise |  |  |  |  |  |  |  |  |  |  |
| C.2 Take time off |  |  |  |  |  |  |  |  |  |  |
| C.3 Self-medicate (alcohol, prescription medications, illegal drugs, etc.) |  |  |  |  |  |  |  |  |  |  |
| C.4 Counselling/Therapy |  |  |  |  |  |  |  |  |  |  |
| C.5 Religious practice |  |  |  |  |  |  |  |  |  |  |
| C.6 Mindfulness exercises |  |  |  |  |  |  |  |  |  |  |
| C.7 Calling support phone lines |  |  |  |  |  |  |  |  |  |  |
| C.8 Peer support |  |  |  |  |  |  |  |  |  |  |
| C.9 Family support |  |  |  |  |  |  |  |  |  |  |
| C.10 Supervisor support |  |  |  |  |  |  |  |  |  |  |
